# Supplementary material for: Variation in Nutritional Components and Antioxidant Capacity of Different Cultivars and Organs of Basella alba
Source: Plants (Basel). 2024 Mar 20;13(6):892. doi: 10.3390/plants13060892 (PMC10974134; doi:10.3390/plants13060892)
Supplement: Supplementary file 1 [file plants-13-00892-s001.zip › Zhang et al. Supplementary Table S2.pdf]

Supplemental Table S2

Correlation of the nutritional components and antioxidant capacity in *Basella alba*.

	Total soluble sugar	Total soluble protein	Total chlorophyll	Total carotenoids	Total ascorbic acid	Total proanthocyanidins	Total flavonoids	Total phenolics	FRAP	ABTS
**Total soluble sugar**	1									
**Total soluble protein**		1								
**Total chlorophyll**			1							
**Total carotenoids**			0.978512	1						
**Total ascorbic acid**					1					
**Total proanthocyanidins**	-0.827309					1				
**Total flavonoids**			0.728157	0.709900			1			
**Total phenolics**					0.690737			1		
**FRAP**	-0.750883					0.817969			1	
**ABTS**	-0.763883					0.682161		0.771312	0.871155	1
